# Band Ligation or Sclerotherapy as Endoscopic Treatment for Oesophageal Varices in Schistosomotic Patients: Results of a Randomized Study

**DOI:** 10.1155/1998/68394

**Published:** 1998-08

**Authors:** Eduardo Sampaio Siqueira, Maria Rachel Da Silveria Rohr, Ermelindo Della Libera, Rubem Robson O. Castro, Angelo Paulo Ferrari

**Affiliations:** 1 Endoscopy Unit Universidade Federal de São Paulo-Escola Paulista de Medicina Division of Gastroenterology Brazil; 2 Rua Machado Bittencourt 379 apt. 91 São Paulo-SP CEP 04044-001 Brazil

## Abstract

Endoscopic sclerotherapy and banding ligation are the two preferred methods to treat oesophageal variceal bleeding. There are many reports dealing with such treatment in cirrhotic patients but we do not know how good they are to treat varices secondary to other forms of portal hypertension. Schistosomiais mansoni is the main cause of portal hypertension and oesophageal varices in Brazil. We performed a prospective randomised study to compare: 1) the efficacy of both treatments in eradicating oesophageal varices, and 2) complications secondary to both treatments. Forty patients were divided in two Groups. Both sclerotherapy and banding ligation were performed until variceal eradication. There were no severe complications. Variceal eradication was faster obtained with banding ligation than sclerotherapy although there was no statistical difference (mean number of sessions 3.05 *vs*  3.72, *p*=0.053). Benign complications were equally frequent in both Groups, although additional sedation was more common in the sclerotherapy Group. We concluded that both treatments are equally effective in the eradication of oesophageal varices, although banding ligation is better tolerated by the patient and probably faster.


*HPB Surgery*, 1998, Vol. 11, pp. 27–32
Reprints available directly from the publisher
Photocopying permitted by license only

© 1998 OPA (Overseas Publishers Association) N.V.
Published by license under
the Harwood Academic Publishers imprint,
part of The Gordon and Breach Publishing Group.
Printed in India.

### Band Ligation or Sclerotherapy as Endoscopic Treatment for Oesophageal Varices in Schistosomotic Patients: Results of a Randomized Study

EDUARDO SAMPAIO SIQUEIRA[a], MARIA RACHEL DA SILVERIA ROHR[a], ERMELINDO DELLA LIBERA[a], RUBEM ROBSON O. CASTRO[a] and ANGELO PAULO FERRARI[b,*]

[a] *Fellow of the Division of Gastroenterology;*
[b] *Director of Endoscopy Unit, Universidade Federal de São Paulo-Escola Paulista de Medicina, Division of Gastroenterology*

*(Received 26 December 1997)*

**Endoscopic sclerotherapy and banding ligation are the two preferred methods to treat oesophageal variceal bleeding. There are many reports dealing with such treatment in cirrhotic patients but we do not know how good they are to treat varices secondary to other forms of portal hypertension. Schistosomiais mansoni is the main cause of portal hypertension and oesophageal varices in Brazil. We performed a prospective randomised study to compare: 1) the efficacy of both treatments in eradicating oesophageal varices, and 2) complications secondary to both treatments. Forty patients were divided in two Groups. Both sclerotherapy and banding ligation were performed until variceal eradication. There were no severe complications. Variceal eradication was faster obtained with banding ligation than sclerotherapy although there was no statistical difference (mean number of sessions 3.05 *vs* 3.72, $p = 0.053$). Benign complications were equally frequent in both Groups, although additional sedation was more common in the sclerotherapy Group. We concluded that both treatments are equally effective in the eradication of oesophageal varices, although banding ligation is better tolerated by the patient and probably faster.**

*Keywords:* Portal hypertension, oesophageal varices, upper gastrointestinal bleeding, schistosomiasis, endoscopic sclerotherapy, oesophageal banding ligation

### INTRODUCTION

Upper gastrointestinal haemorrhage (UGH) secondary to oesophageal varices (EV) rupture is a severe clinical complication of portal hypertension (PH) [1]. It occurs in approximately 30% of patients with hepatic cirrhosis and EV [2, 3]. First episode mortality rate is high, up to 50% of patients [3, 4]. Re-bleeding in non treated patients occurs in 47% to 84% of cases and the correspondent mortality rate ranges from 20% to 70% [5, 6].

Sclerotherapy (ST) and band ligation (BL) are the two most widely used endoscopic procedures in the treatment of EV [7].

Endoscopic sclerotherapy shows up to 95% efficacy in the treatment of a severe variceal bleeding episode [5, 8]. Endoscopic treatment should be the choice for re-bleeding prophylaxis, if liver transplantation is not available. ST should be considered as prophylaxis for the first variceal bleeding episode in patients with high

*Author for correspondence: Dr. Angelo P. Ferrari, Rua Machado Bittencourt, 379 apt. 91, CEP 04044-001, São Paulo-SP-Brazil.

bleeding risk and reasonable life expectancy [4, 7]. However, there is a high incidence of complications that may reach 70% of procedures [9].

By eliminating sclerosant solutions, endoscopic banding ligation has reduced the complication rate associated with EV endoscopic treatment [10–12]. Several prospective studies have proven the efficacy of such procedure in the treatment of EV bleeding [13, 14].

Most studies on PH and EV were conducted in patients with hepatic cirrhosis of several aetiologies (alcoholic, viral, auto-immune). Studies in patients with other types of portal hypertension, such as schistosomiasis, are less frequently found.

Schistosomiasis mansoni leads to pre-sinusoidal PH and preserved hepatic tissue [15–17], and is the major cause for PH and variceal bleeding in Brazil. As a result of a reasonably sustained liver function, hepatosplenic schistosomotic (HSS) patients live longer and better as compared to cirrhotic, and variceal UGH is their main clinical manifestation [18]. A number of studies have proven the efficacy of ST and surgical procedures (shunts and devascularization) in preventing re-bleeding in such patients [19–21]. The few studies using primary prophylaxis were not conclusive [15, 16]. As far as we are concerned this is the first study on the use of BL for oesophageal varices treatment in HSS patients.

We conducted a randomised prospective study comparing the efficacy and complications of ST and BL in the treatment of EV in schistosomotic patients. Variceal recurrence was assessed over a six-month period.

### MATERIAL AND METHODS

From August 1994 to August 1995, schistosomotic patients over 18 years of age, who had medium (F2) or large size (F3) oesophageal varices (according to the Japanese Research Society for Portal Hypertension criteria [22]) were included in the study. Informed consent was obtained from all patients and the study was approved by UNIFESP-Hospital São Paulo Ethics Committee. Aetiology of liver disease was based on abdominal ultrasound findings, specific for HSS [23, 24]. Patients with positive serology for hepatitis B (HBsAg) and C virus (anti-HCV) as well as those with history of alcohol intake in the past were excluded.

Patients were randomised into two Groups by means of sealed envelopes. Group 1 comprised patients who underwent sclerotherapy treatment and Group 2 those submitted to banding ligation. There were 20 patients in each Group.

### Endoscopic Treatment

Endoscopic procedures were performed by experienced gastroenterologists, using a Olympus GIF-V10 endoscope. All patients were sedated with 50 mg of meperidine IV. All patients, except, 3 were treated electively.

We performed predominantly intra-variceal injections of 2.5% ethanolamine oleate (Glaxo-Brasil), through a 7 Fr diameter injection catheter with a 4 mm/23 G needle ( Wilson-Cook Medical Inc., USA). Initial injections were performed around the cardia. Maximum volume of sclerosant was 5 ml/site and 35 ml/session.

Banding ligation was performed according to usual techniques following the introduction of a 20 cm plastic overtube over an oesophageal dilator (Savary-Gillard), described by Stiegmann [11], with commercial kits (Bard Inc., USA). The first O-ring was placed in the distal parts, and more proximal O-rings also were used in long varices. The number of O-rings used per session was not greater than 5.

### Follow-up

Sclerotherapy and BL sessions were repeated at intervals ranging from 10 to 20 days until eradication of all oesophageal varices. Sessions were postponed if large lesions were present. Patients who had bleeding episodes over the course of treatment or even after eradication were hospitalised and underwent urgent endoscopy for diagnosis of the bleeding site and possible endoscopic treatment. Patients were referred to surgery if endoscopy was unsuccessful.

The study was completed six months after eradication, and at that point another endoscopy was performed to look for variceal recurrence.

### Statistical Analysis

Mann-Whitney test was used to compare means. All operations were computer analysed (Primer of Statistics, v 3.02, McGraw-Hill, Inc.,) In all tests $p$ value was established as 5% or 0.05.

TABLE I Feature of the 40 patients previously to the study

| Features | Group 1 (ES) N=20 | Group 2 (BL) N=20 |
|---|---|---|
| Sex (M/F) | 8/12 | 10/10 |
| Age | | |
| Range | 24 – 70 | 23 – 64 |
| Mean | 41.95 | 42.65 |
| Median | 39 | 43 |
| PT (min)* | 1.34 ± 0.13 | 1.34 ± 0.16 |
| Albumin (g/dl)* | 3.47 ± 0.45 | 3.81 ± 0.88 |
| AST (u/l)* | 15.7 ± 4.4 | 16.9 ± 5.6 |
| ALT (u/l)* | 16.5 ± 6.3 | 19.1 ± 7.9 |
| γ GT (u/l)* | 15.2 ± 4.4 | 17.6 ± 9.3 |
| Previous surgery | 4 | 2 |
| Sclerotherapy | 3 | 4 |
| Previous UGH | 6 | 6 |
| LV | 17 | 16 |
| RS | 14 | 13 |
| GV | 4 | 3 |
| Gastropathy | 4 | 3 |

* Laboratory figures express the Groups mean ± standard deviation. LV = large size varices; RS = red signs; GV = gastric varices; Previous surgery = portal hypertension surgery; Gastropathy = severe congestive gastropathy at index endoscopy.

### RESULTS

Forty patients were enrolled (20 in each Group); 39 returned for the 6 month control examination. Two patients underwent surgery for uncontrolled UGH due to gastric variceal rupture, prior to EV eradication.

Both Groups were similar regarding sex, age and laboratory tests (Tab. I). Endoscopic findings and data concerning digestive bleeding and portal hypertension treatment before the study showed no significant difference in either Group (Tab. I).

One patient from Group 1 had ascites at the time of randomisation. All others had compensated HSS. All patients had peri-portal thickness (fibrosis) at abdominal ultrasound examination.

All 123 treatment sessions were done in an elective mode, except for 3 patients (2 in Group 1 and one in Group 2) in whom the first session was performed as urgent therapy.

### Efficacy

In Group 1, before varices were eradicated, two patients had uncontrolled bleeding from fundic gastric varices and were referred to surgery. One died of kidney failure and disseminated intravascular coagulation two days after surgery. Efficacy of endoscopic treatment was 90%. After excluding the two operated patients, the number of sessions required for eradication ranged from 2 to 7 (mean: 3.72). There was no significant difference when the number of sessions required to obliterate the varices was compared in both Groups, however there was a tendency to faster eradication in Group 2 ($p = 0.053$, Mann-Whitney test) as shown in Table II. All patients in Group 2 had their EV eradicated after 1 to 5 sessions (mean 3.05, 100% efficacy). No variceal bleeding was observed during treatment.

One patient in Group 1 had one UGH episode due to EV rupture (recurrence) before the end of treatment, with moderate hemodynamic repercussions. This episode was successfully managed by endoscopy. The same patient had another UGH episode four months after EV eradication. Endoscopic evaluation revealed congestive gastropathy as the source of bleeding. Beta-blocker treatment was initiated.

TABLE II Treatment results for both Groups

| Results | Group 1 (ST) N = 18* | Group 2 (BL) N = 20 |
|---|---|---|
| Sessions before eradication | | |
| Total | 67 | 61 |
| Mean** | 3.72 | 3.05 |
| Range | 2 – 7 | 1 – 5 |
| Sclerosant volume | | |
| mean/session | 15.4 ml | |
| mean/patient | 61.7 ml | |
| Rings | | |
| mean/patient | | 10.7 |
| mean/session | | 3.6 |
| Eradication rate (%) | 90 | 100 |
| Mortality | 1/20 (5%) | – |

* values for all items, except mortality, refer to patients in whom variceal eradication was achieved.
** $p = 0.053$ (Mann-Whitney test).

### Recurrences

No oesophageal recurrence was found in Group 2. One patient in Group 1 had variceal recurrence 6 months after eradication. Varices were again treated and eradicated with three ST sessions.

### Complications

There was no oesophageal perforation or infection in either Group. Massive bleeding after injection occurred in four procedures in Group 1 (5.0%), and all were controlled. Although no such complication occurred in Group 2, the difference between both Groups was not statistically significant.

Complaints of dysphagia, odynophagia and thoracic pain of different intensity and duration in the days following the procedures were reported by nearly all patients, with no significant difference between the Groups. Nine patients in Group 1 (45%) required additional sedation during or at the end of the procedure due to intense thoracic pain. Among such patients, 18/77 (23.38%) procedures needed additional sedation, all from Group 1.

Overtube placement did not cause any extra complaint. At the final examination, 3 patients (15%) from Group 1 and 2 from Group 2 (10%) showed slight asymptomatic narrowing of the oesophageal lumen without the necessity of any therapeutic approach.

Oesophageal membrane injuries (erosions or ulcerations) were found in all patients. Ulcerations secondary to sclerotherapy were deeper and more irregular than after BL. Ulcerations did not cause any episode of bleeding, however sessions had to be postponed in 11 (14.28%) procedures in Group 1 and 3 (5.1%) in Group 2 ($p = 0.147$).

Mucosa scars and neo-vascularization were often noted at the end of treatment. Scars were more severe in patients in Group 1. Mucosal bridges were present in two asymptomatic patients in Group 1. As mentioned above, one patient in Group 1 developed congestive gastropathy, leading to a digestive bleeding episode.

### DISCUSSION

The mortality rate in cirrhosis due to UGH following EV rupture is high, reaching up to 50% of patients [1, 25]. In Brazil the major cause of such complication is HSS, and patients have a better outcome as compared to other aetiologies [17]. The natural history of HSS is not fully understand. The estimate number of patients developing EV is not available nor variceal bleeding episodes that may occur in the course of the disease, or the mortality rate in bleeding episodes. Moreover, studies conducted in this Group of patients are less frequent than those concerning cirrhosis, making it impossible to assert that the results of the treatment for cirrhosis tally those for schistosomiasis.

Although surgical treatment is largely used in HSS patients with bleeding EV [15, 21, 26, 27], endoscopic treatment can also be successfully used [19, 20, 28]. Data on the role of ST or any other prophylactic treatment for the first bleeding episode in schistosomotic patients are limited.

Our study compared two endoscopic procedures (ST and BL) for EV treatment in schistosomotic patients. According to our literature review, this is the first study to describe the use of BL in such patients. In the present study, ST efficacy was confirmed , and eradication rate was 90%.

The average number of ST sessions required to eradication (3, 72) was similar to the published data, both for cirrhosis and schistosomiasis, although only patients with medium and large size varices were enrolled in this study [13, 14, 19]. No EV bleeding was noted eradication. Variceal recurrence after sclerotherapy treatment reaches upto 40% in the first year following treatment [1, 19, 29]. Our recurrence rate after 6 months was 5%.

Incidence of post ST complications was also similar to those reported in the literature [14, 19]. There were no episodes of severe complications such as oesophageal perforation, infection and oesophageal symptomatic stricture. Bleeding episodes triggered during the procedures occurred in 5.1% of the cases. Despite the presence of mucosal lesions (erosions and ulcerations) in all patients, symptoms reported by the patients during follow-up (dysphagia, odynophagia and thoracic pain) cannot be imputed to them, because at the time of the assessment they were already asymptomatic and lesions were still present. Such complaints were self-limited, and required no medication. In 3.1% of the procedures, the use of additional sedation was necessary, this figure is higher than reported in the literature.

In the present study, BL showed efficacy of 100%. The average number of sessions required to eradicate the varices was 3.05, smaller than patients submitted to ST (3.72; $p = 0.053$). Should there be an increase in the sample size, significance would probably be obtained. Besides, in the present study a maximum of five O-rings/session was established, and using more O-rings/session could lead to a reduction in the number of sessions. That approach does not increase complications, as observed in other patients treated in our Unit and not enrolled in the study.

In this Group, acute mucosal lesions (erosions and ulcerations) were also described in all patients. Although asymptomatic such lesions accounted for some of the postponement of new sessions in both Groups. All lesions healed spontaneously.

Mucosal scars were also described in most patients. Both acute (erosions and ulcerations) and chronic (scars, neo-vascularization) lesions were less severe in the BL Group.

Occurrence of only one episode of digestive bleeding due to congestive gastropathy in the ST Group does not entitle us to state that such complication occurs more often in this Group.

Incidence of variceal recurrence following BL is yet to be defined. Some authors claim it to occur more often in cirrhotic patients when compared to ST [30]. No cases of EV recurrence had been noticed in our study, up to six months following variceal eradication. patients from both Groups are still being followed up for detection of late recurrences.

At the end of the study, the efficacy and safety of BL as EV treatment in HSS patients were confirmed. When compared to ST, BL was less painful and maybe faster in EV eradication.

### *Acknowledgments*

This study was in part supported by a grant from FAPESP, process #93/3398-0.

### *References*

[1] Sherlock, S. (1990). Oesophageal varices. *American Journal of Surgery*, **160**, 9–12.

[2] Kleber, G., Sauerbruch, T., Ansari, H. and Paumgartner, G. (1991). Prediction of variceal hemorrhage in cirrhosis: a prospective follow-up study. *Gastroenterology*, **100**, 1332–1337.

[3] Mccormick, P. A. (1994). Pathophysiology and prognosis of oesophageal varices. *Scandinavian Journal Gastroenterology*, **29** (Suppl. 207) 1–5.

[4] Sauerbruch, T. (1994). Prophylaxis of first variceal bleeding: where does the truth lie? *Endoscopy*, **26**, 748–749.
[5] Krige, J. E. J. and Bornman, P. C. (1992). The treatment of oesophageal varices. *Annual Revision of Medicine*, **43**, 69–82.
[6] D'amico, G., Pagliaro, L. and Bosch, J. (1995). The treatment of portal hypertension: a meta-analytic review. *Hepatology*, **22**, 332–353.
[7] Terblanche, J. (1992). Issues in gastrointestinal endoscopy: oesophageal varices: inject, band, medicate or operate. *Scandinavian Journal of Gastroenterology*, **27** (Suppl. 192) 63–66.
[8] Larson, A. W., Cohen, H., Zweiban, B., Chapman, D., Gourdji, M., Korula, J. and Weiner, J. (1986). Acute oesophageal variceal sclerotherapy. *Jama*, **255**, 497–500.
[9] Schuman, B. M., Beckman, J. W., Tedesco, F. J., Griffin, J. W. J. and Assad, R. T. (1987). Complications of endoscopic injection sclerotherapy: a review. *American Journal of Gastroenterology*, **82**, 823–830.
[10] Stiegmann, G. V., Cambre, T. and Sun, J. H. (1986). A new endoscopic elastic band ligation device. *Gastrointestinal Endoscopy*, **32**, 230–233.
[11] Stiegmann, G. V. and Goff, J. S. (1988). Endoscopic varix ligation (ELV): preliminary clinical experience. *Gastrointestinal Endoscopy*, **34**, 113–118.
[12] Stiegmann, G. V., Goff, J. S., Sun, J. H., Davis, D. and Bozdech, J. (1989). Endoscopic variceal ligation: an alternative to sclerotherapy. *Gastrointestinal Endoscopy*, **35**, 431–434.
[13] Stiegmann, G. V., Goff, J. S., Michabltz-Onody, P. A., Korula, J., Lieberman, D., Saeed, Z. A., Reevilbl, R. M., Sun, J. H. and Lowenstein, S. R. (1992). Endoscopic sclerotherapy as compared with endoscopic ligation for bleeding oesophageal varices. *The New England Journal of Medicine*, **326**, 1527–1532.
[14] Laine, L. and Cook, D. (1995). Endoscopic ligation compared with sclerotherapy for treatment of oesophageal variceal bleeding. A meta-analysis. *Annals of Internal Medicine*, **123**, 280–287.
[15] Raia, S., Mies, S. and Alfieri, F. J. (1991). Portal hypertension in mansonic schistossomiasis. *World Journal of Surgery*, **15**, 176–187.
[16] Paes, I. B. (1991). Escleroterapia profilática das varizes esofagogástricas: existem evidencias de sua eficácia? In: L. P. Castro, P. R. S. Rocha and A. S. Cunha, *Tápicos em Gastroenterologia*, **2**, 125–130 Rio de Janeiro, Medsi.
[17] Da Silva, L. C. and Carrilho, F. J. (1992). Hepatosplenic schistosomiasis-pathophysiology and treatment. *Gastroenterological Clinics of North America*, **21**, 163–177.
[18] McCormick, P. A. and Burroughs, A. K. (1994). Relation between liver pathology and prognosis in patients with portal hypertension. *World Journal of Surgery*, **16**, 171–174.
[19] Cordeiro, F. (1990). Variceal sclerosis in schistosomotic patients: a 5-year follow study. *Gastrointestinal Endoscopy*, **36**, 475–478.
[20] Sakai, P., Boaevntura, S., Ishioka, S., Mies, S., Sette, H. J. and Pinotti, H. W. (1990). Sclerotherapy of bleeding oesophageal varices in schistosomiasis. *Endoscopy*, **22**, 5–7.
[21] Raia, S., Gayotto, L. C. C., Forster, S. C., Fukushima, J. and Strauss, E. (1994). Portal hypertension is schistosomiasis: a long-term follow-up of a randomised trial comparing three types of surgery. *Hepatology*, **20**, 398–403.
[22] Japanese Research Society For Portal Hypertension (1980). The general rules for recording endoscopic findings on oesophageal varices. *Japanese Journal of Surgery*, **10**, 84–87.
[23] Cerri, G. G., Ablvs, V. A. F. and Magalhães, A. (1984). Hepatosplenic schistosomiasis mansoni: ultrasound manifestations. *Radiology*, **153**, 777–780.
[24] Hatz, C., Jenkins, J. M., Ali, Q. M., Abdel-Wahab, M. F., Cerri, G. G. and Tanner, M. (1992). A review of the literature on the use of ultrasonography in schistosomiasis with special reference to its use in field studies. *Acta Tropical*, **51**, 15–28.
[25] Merkel, C., Massimo, B., Angeli, P., Noevnta, F., Caregaro, L., Sacerdoti, D. and Gatta, A. (1989). Prognostic indicators of survival in patients with cirrhosis and oesophageal varices without previous bleeding. *American Journal of Gastroenterology*, **84**, 717–722.
[26] Salam, A. A., Ezzat, F. A. and Abu-Elmagd, K. M. (1990). Selective shunt in schistosomiasis in Egypt. *American Journal of Surgery*, **160**, 90–93.
[27] Conn, H. O. (1994). A randomised comparison of three types of surgery in schistosomal portal hypertension many fewer answers than questions. *Hepatology*, **20**, 526–528.
[28] El-Zayadi, A., El-Din, S. S. and Kabil, S. M. (1988). Endoscopic sclerotherapy versus medical treatment for bleeding oesophageal varices in patients with schistosomal liver disease. *Gastrointestinal Endoscopy*, **34**, 314–317.
[29] Parikh, S. S. and Desai, H. G. (1992). What is the aim of oesophageal variceal sclerotherapy-prepisodeion of rebleeding or complete eradication of veis? *Journal of Clinical. Gastroenterology*, **15**, 186–188.
[30] Hou, M., Lin, H., Kuo, B. I., Chen, C., Ble, F. and Ble, S. (1995). Comparison of endoscopic variceal injection sclerotherapy and ligation for the treatment of oesophageal variceal hemorrhage: a prospective randomised trial. *Hepatology*, **21**, 1517–1522.

